# Morphology-adaptive total variation for the reconstruction of quantitative susceptibility map from the magnetic resonance imaging phase

**DOI:** 10.1371/journal.pone.0196922

**Published:** 2018-05-08

**Authors:** Li Guo, Yingjie Mei, Jijing Guan, Xiangliang Tan, Yikai Xu, Wufan Chen, Qianjin Feng, Yanqiu Feng

**Affiliations:** 1 Guangdong Provincial Key Laboratory of Medical Image Processing, School of Biomedical Engineering, Southern Medical University, Guangzhou, China; 2 Philips Healthcare, Guangzhou, China; 3 Department of Medical Imaging Center, Southern Medical University Nanfang Hospital, Guangzhou, China; UNITED STATES

## Abstract

Quantitative susceptibility mapping (QSM) is a magnetic resonance imaging technique that quantifies the magnetic susceptibility distribution within biological tissues. QSM calculates the underlying magnetic susceptibility by deconvolving the tissue magnetic field map with a unit dipole kernel. However, this deconvolution problem is ill-posed. The morphology enabled dipole inversion (MEDI) introduces total variation (TV) to regularize the susceptibility reconstruction. However, MEDI results still contain artifacts near tissue boundaries because MEDI only imposes TV constraint on voxels inside smooth regions. We introduce a Morphology-Adaptive TV (MATV) for improving TV-regularized QSM. The MATV method first classifies imaging target into smooth and nonsmooth regions by thresholding magnitude gradients. In the dipole inversion for QSM, the TV regularization weights are a monotonically decreasing function of magnitude gradients. Thus, voxels inside smooth regions are assigned with larger weights than those in nonsmooth regions. Using phantom and in vivo datasets, we compared the performance of MATV with that of MEDI. MATV results had better visual quality than MEDI results, especially near tissue boundaries. Preliminary brain imaging results illustrated that MATV has potential to improve the reconstruction of regions near tissue boundaries.

## Introduction

Magnetic susceptibility is a fundamental physical property that describes the response of biological tissues to an applied magnetic field. The magnetic susceptibility inhomogeneity field map may be measured from the magnetic resonance imaging (MRI) phase data [1]. In quantitative susceptibility mapping (QSM), tissue magnetic susceptibility distribution is determined by deconvolving the local tissue field map with a dipole kernel [1–5]. Given the zero values of the dipole kernel along the magic angle in the **k**-space, the inversion of the local tissue field map to the tissue magnetic susceptibility distribution is an ill-conditioned problem that causes streaking artifacts and amplifies noise in reconstructed susceptibility maps [6, 7].

To achieve accurate susceptibility reconstruction, one approach is to collect phase data at multiple head orientations with respect to the main magnetic field and calculate susceptibility maps by using methods such as the calculation of susceptibility through multiple orientation sampling (COSMOS) [8] and the susceptibility tensor imaging (STI) [9]. Multiple orientation sampling is not clinically feasible for QSM because it substantially prolongs scan time. Moreover, the repositioning of imaging subjects in a fixed magnet is restricted to a narrow range in multiple orientation methods. Therefore, the reconstruction of susceptibility maps from single orientation phase data is the primary approach in practice. The results obtained by COSMOS or STI from multiple orientation phase data are the references for evaluating the performance of single orientation methods.

Compared with multiple orientation techniques, single orientation QSM has the advantage of reduced scan time but suffers from streaking artifacts and noise amplification due to the ill-posedness of the inversion problem. To mitigate artifacts and noise, various QSM methods have been developed to address dipole inversion from single orientation sampling [10–25]. Among Bayesian regularization approaches, morphology enabled dipole inversion (MEDI) [11, 12, 26] combine total variation (TV) and morphological information in magnitude to regularize the susceptibility reconstruction. However, the susceptibility maps generated by MEDI may still contain artifacts near tissue edges because it imposes no constraints in these regions.

Here, we introduce a Morphology-Adaptive TV (MATV) regularization method for single orientation QSM to improve the susceptibility reconstruction in regions with tissue edges. The MATV method enforces the TV penalty on the whole susceptibility map and the TV penalty weights are a monotonically decreasing function of magnitude gradient maps. Small regularization weights are added to nonsmooth regions and large regularization weights are added to smooth regions. Gadolinium phantom and in vivo experiments were performed to evaluate the performance of MATV.

## Methods

### Relation between magnetic susceptibility and field

The tissue magnetic susceptibility *χ* is a measure for the amount of magnetization induced in tissue that is exposed to the main magnetic field of an MRI scanner. Convolving *χ* with the z-component of the dipole kernel yields the local tissue magnetic field, *φ*, the change in magnetic susceptibility relative to the main magnetic field. In **r**-space, the susceptibility distribution is defined by the linear relation below:

φ(r)=d(r)*χ(r) (1)

where r is the spatial (**r**-space) location, $d(r)=(\frac{1}{4\pi }\frac{3\cos^{2}\theta -1}{r^{3}})(r\neq 0)$ is the dipole kernel in **r**-space, θ is the azimuthal angle in spherical coordinate, and * is the convolution.

Given a tissue field map, the susceptibility distribution can be obtained by solving Eq (1) in the least-squares sense as follows:

$$\chi ={\mathrm{argmin}}_{\chi }{\left\|F^{H}\mathrm{DF}\chi -\varphi \right\|}_{2}^{2}$$ (2)

Here, *χ* and *φ* are the vector forms of the susceptibility distribution and the local field in the **r**-space, respectively, F is the diagonal matrix form of the Fourier transform operator, $D(k)=(\frac{1}{3}-\frac{k_{z}^{2}}{k^{2}})(k\neq 0)$ is the diagonal matrix form of the dipole kernel in **k**-space, k is the **k**-space vector, and k_z_ is the z-component of k. The magnitude image can be introduced into Eq (2) to penalize noise variation in the field measurement using a weighted least-squares approach as shown below [10]:

$$\chi ={\mathrm{argmin}}_{\chi }{\left\|W_{m}(F^{H}\mathrm{DF}\chi -\varphi )\right\|}_{2}^{2}$$ (3)

where m is the MRI magnitude image, W_m_ is a weighting matrix with entries that are proportional to image magnitude.

Given the zero eigenvalues of the matrix F^H^DF, the minimization problem in Eq (3) does not possess a unique solution. Hence, prior information is exploited to derive a unique estimation of the susceptibility map.

### Solution with MATV

MATV imposes TV regularization on both smooth and nonsmooth regions in the susceptibility map, with different regularization weights in the two regions. The reconstruction of the quantitative susceptibility map via the MATV approach can be formulated as follows:

$$\chi ={\mathrm{argmin}}_{\chi }{\left\|W_{m}(F^{H}\mathrm{DF}\chi -\varphi )\right\|}_{2}^{2}+\lambda {\left\|W_{\nabla m}\nabla \chi \right\|}_{1}$$ (4)

where λ is the regularization parameter that balances the trade-off between the fidelity term and the MATV regularization term, ∇ is the three-dimensional (3D) forward differencing operator[27], W_∇m_ is the weighting diagonal matrix obtained from the gradient maps of the magnitude image and assigns different weights to the x, y, and z-components of ∇χ, and ‖∇*χ*‖_1_ is the L1 norm of the gradient, i.e., TV.

Eq (4) provides a Morphology-Adaptive TV regularization for QSM inversion. In MATV, the TV regularization weights on voxels near edges are determined on the basis of local morphology information calculated from magnitude gradient maps. To avoid the oversmoothness of edges, MATV imposes a continuous edge weighting matrix inversely proportional to magnitude gradient on voxels near edges. Specifically, the weighting matrix W_∇m_ of voxels near edges are assigned to $\sin\left(\frac{\pi }{2}\frac{C}{\left|\nabla m\right|}\right)$, where the threshold c is determined in the same way as in MEDI such that approximately 30% of the voxels in the gradient maps of magnitude image are considered as boundary regions. In all, the weighting matrix is formulated as: $W_{\nabla m}=\left\{ \begin{array}{c} \;1,\;|\nabla m|<\;c\\ \;\sin\left(\frac{\pi }{2}\frac{C}{\left|\nabla m\right|}\right),\;else \end{array}\right.$. Here, similar to MEDI, the TV weights on voxels inside smooth regions (|∇m| <*c*) are set to 1. The TV weights on voxels near edges (|∇m| ≥ *c*) are set to values that are determined by magnitude gradients.

The proposed MATV method differs from MEDI in the following aspect. The TV weight in MEDI can be considered as a hard thresholding function of magnitude gradient. The proposed MATV method determines the weight of TV as a monotonically decreasing function of local gradient in the magnitude images, i.e., the TV weight in MATV is a soft thresholding function of magnitude gradient. In regions near tissue boundaries, MEDI imposes no TV constraint while MATV can still enforce TV constraint but with a reduced degree than that in smooth regions.

### Datasets for evaluation

In this study, gadolinium phantom and in vivo datasets were used to assess the performance of MATV. The associated imaging parameters are briefly described below.

#### Gadolinium phantom

The phantom dataset was downloaded from the Cornell University website (http://weill.cornell.edu/mri/QSM/Online.zip). An agarose (2%) gel phantom that contained five balloons filled with gadolinium solution (Magnevist, Berlex Labrotories, Wayne, NJ) was prepared. The expected susceptibility values of 0.05, 0.1, 0.2, 0.4, and 0.8 part per million (ppm) were assigned to the five gadolinium balloons. MRI imaging was conducted on a 3 Tesla (T) scanner (GE, Waukesha, WI) with a multiecho gradient echo (GRE) sequence. The scan parameters were as follows: flip angle (FA) = 15°, multiple echo times (TEs) = 5, 8.4, 11.8, 15.2, 18.6, 22, 25.4, 28.8 ms, repetition time (TR) = 70 ms, bandwidth (BW) = 480 Hz/pixel, matrix size = 130×130×116, and isotropic resolution = 1×1×1 mm^3^. 12 different orientations with rotational angles that varied from 40° to 140° relative to the main field were acquired.

#### In vivo data

In this study, two in vivo brain datasets were used. The in vivo dataset 1 was downloaded from the Cornell University website (http://weill.cornell.edu/mri/QSM/Online.zip) and the in vivo dataset 2 was downloaded from the QSM reconstruction challenge website (http://qsm.neuroimaging.at) [28].

The in vivo dataset 1 of a healthy volunteer was also used in previously published study [29]. This dataset was imaged on a 3 T scanner (HDx, GE, Waukesha, WI) with multiecho GRE sequence. The imaging parameters were as follows: FA = 15°, TEs = 5, 10, 15, 20, 25, 30, 35, 40, 45, 50 ms, TR = 55 ms, BW = 260 Hz/pixel, matrix size = 256×256×146, voxel size = 0.9375×0.9375×1 mm^3^, and R = 2. Five different orientations with rotational angles varied from 15° to 35° relative to the main field were acquired.

The in vivo dataset 2 of a healthy volunteer was also used in previously published study [30]. This 3D-GRE dataset was scanned on a 3 T system (Siemens Tim Trio) with wave controlled aliasing in parallel imaging (Wave-CAIPI) sequence. The imaging parameters included [30]: FA = 14°, TE = 25 ms, TR = 35 ms, BW = 100 Hz/pixel, matrix size = 240×240×168, isotropic resolution = 1.1×1.1×1.1 mm^3^, and parallel imaging acceleration factor R = 15. 12 head orientations with maximum rotational angles = 25.4° relative to the main static field were acquired.

### Implementation and evaluation

For the phantom and in vivo brain 1, the field map and COSMOS reference map are downloaded from the MEDI package (http://weill.cornell.edu/mri/QSM/Online.zip). The detailed processing steps are as follows: the field map was computed from the multiecho GRE datasets using a nonlinear fitting approach followed by a magnitude image guided phase unwrapping method [31], where the magnitude image was calculated by taking the square root of the sum of the squares of all the GRE echoes. The background field was removed by the projection onto dipole field algorithm (PDF) [32]. The COSMOS reference data was also preprocessed with the same pipeline. The coregistration required by COSMOS was accomplished using FMRIB’s Linear Image Registration Tool (FLIRT) [33] with the first orientation used as the reference. For the in vivo brain 2, the field map and COSMOS reference map are downloaded from the QSM reconstruction challenge website (http://qsm.neuroimaging.at). The detailed processing steps are as follows: the Laplacian unwrapping method [34] was used for phase unwrapping and the Laplacian Boundary Value (LBV) method [35] was used for background field removal. The reference susceptibility map of COSMOS was chosen as reference for in vivo brain 2 and computed with the same preprocessing pipeline. The coregistration required by COSMOS was accomplished using FLIRT with the neutral orientation (coincide with the main magnetic field) used as the reference scan orientation.

For comparison, the downloaded MEDI code was implemented with the use of forward difference in the calculation of gradient. The regularization parameters of MEDI were set to the same values as in previous published papers [29, 36]. The regularization parameters for MATV in the phantom and brain were individually determined through visual inspection and the quantitative metrics, i.e., RMSE, SSIM, HFEN, and regression slope. The regularization parameters in MATV were selected as follows: λ = 0.0017 in the gadolinium phantom, λ = 0.0007 in brain 1 and 2.

The accuracy of susceptibility reconstruction was assessed by calculating the relative mean square error (RMSE) 100‖*χ*_recon_ − *χ*_ref_‖_2_/‖*χ*_ref_‖_2_ between the estimated susceptibility *χ*_recon_ and the reference susceptibility *χ*_ref_. Besides RMSE, high frequency error norm (HFEN) [37], and structure similarity index (SSIM) [38] with respect to the reference susceptibility map were also employed to evaluate the performance of MATV. For the phantom dataset, the linear regression analysis of MATV and MEDI with respect to COSMOS was performed. The slope and coefficient of determination (R^2^) from the regression analysis of MEDI are consistent with the one reported previous paper[29].

All the computations were performed using MATLAB programming environment (MathWorks, Natick, MA). The MATLAB codes were implemented on a computer with 3.20 GHz CPU and 8 GB RAM. The MATLAB code of the proposed MATV algorithm can be downloaded from https://ww2.mathworks.cn/matlabcentral/fileexchange/67079-the-matlab-code-for-matv-algorithm.

## Results

### Gadolinium phantom

**Fig 1:**
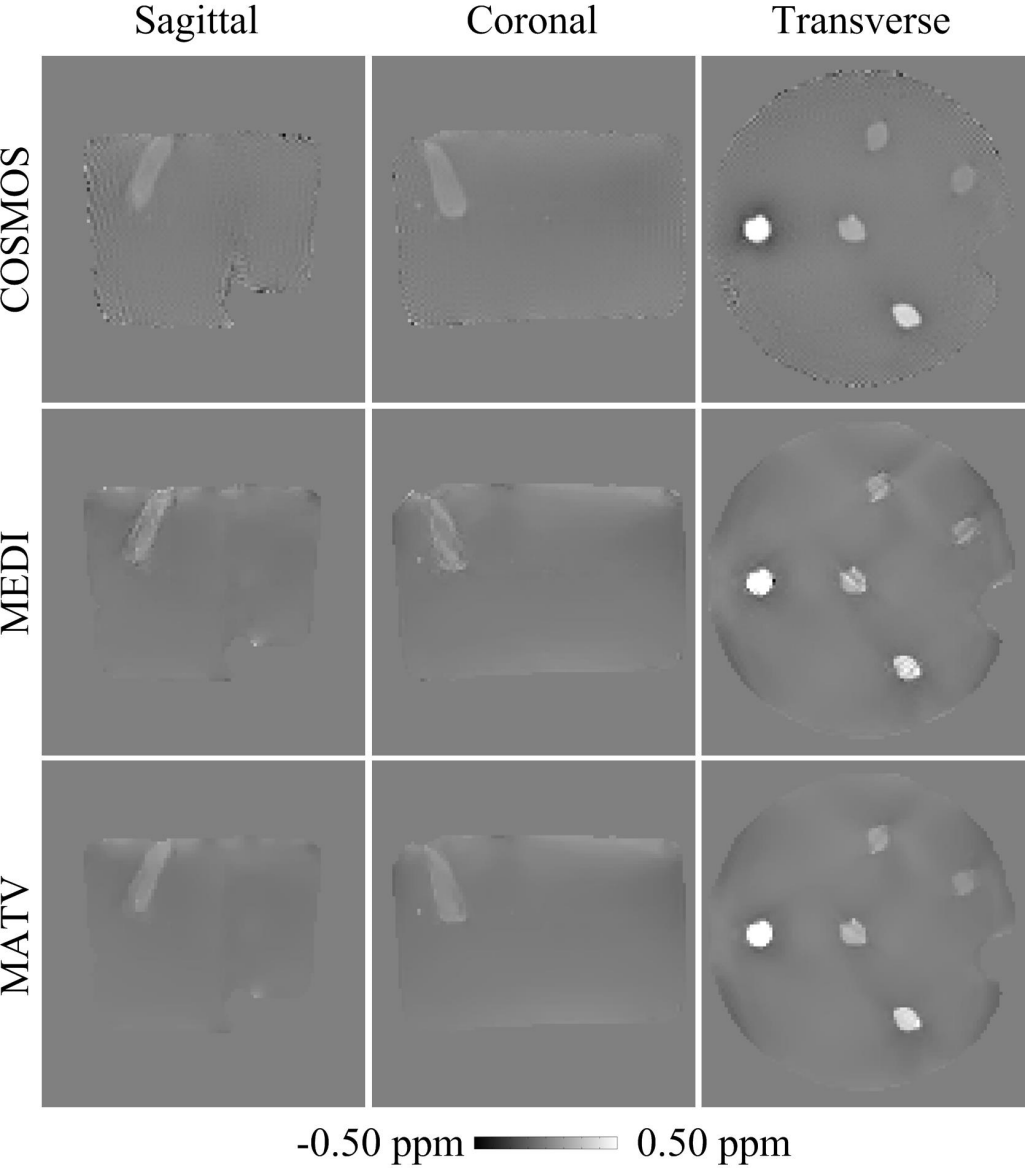
QSM reconstructions for the phantom dataset. First row: sagittal, coronal, and transverse views of susceptibility maps reconstructed by COSMOS. Second row: sagittal, coronal, and transverse views of susceptibility maps reconstructed by MEDI. Third row: sagittal, coronal, and transverse views of susceptibility maps reconstructed by MATV.

**Fig 2:**
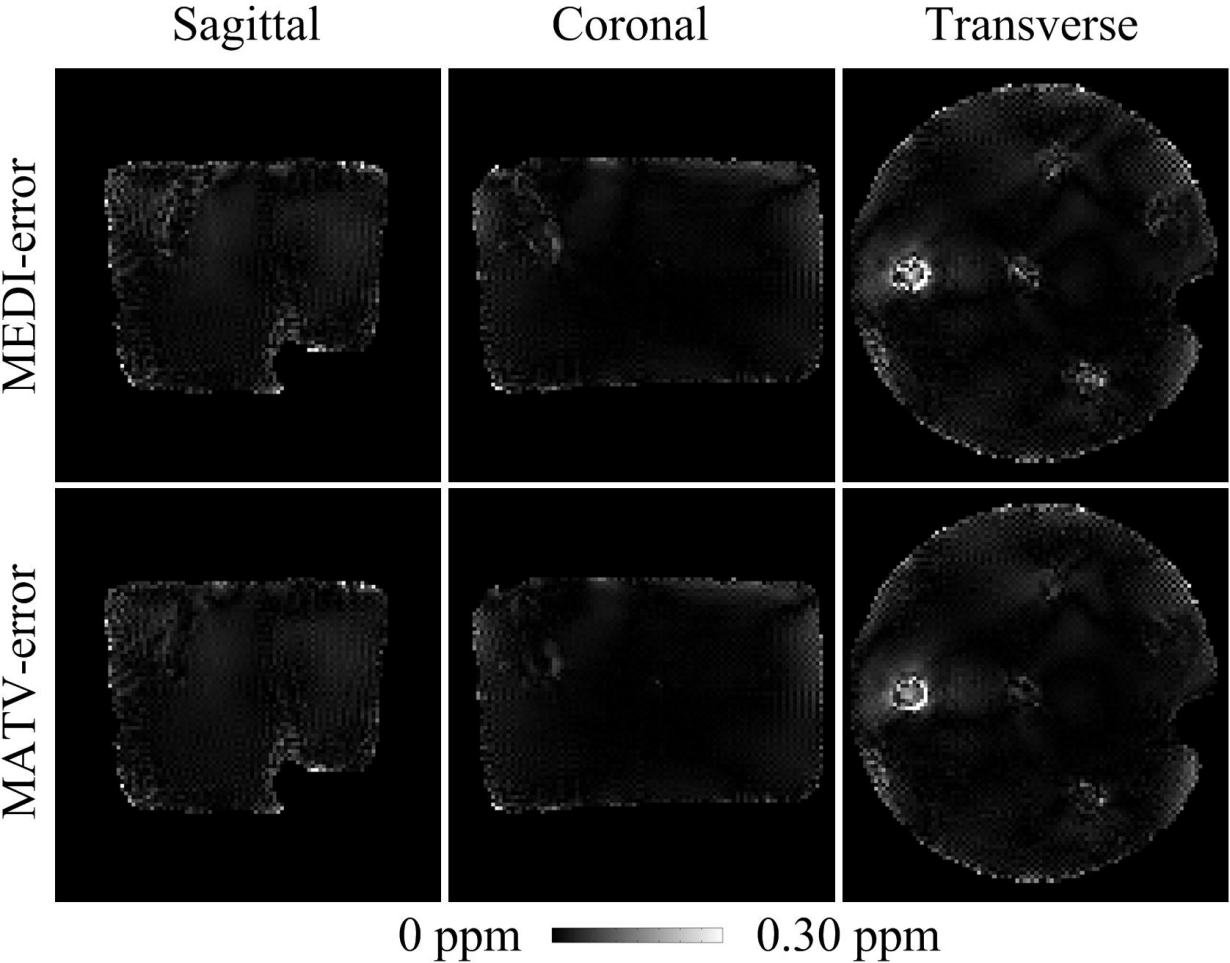
Absolute error maps for the phantom dataset. The susceptibility maps determined by COSMOS were regarded as references. First row: sagittal, coronal, and transverse views of absolute error maps of MEDI. Second row: sagittal, coronal, and transverse views of absolute error maps of MATV.

**Fig 3:**
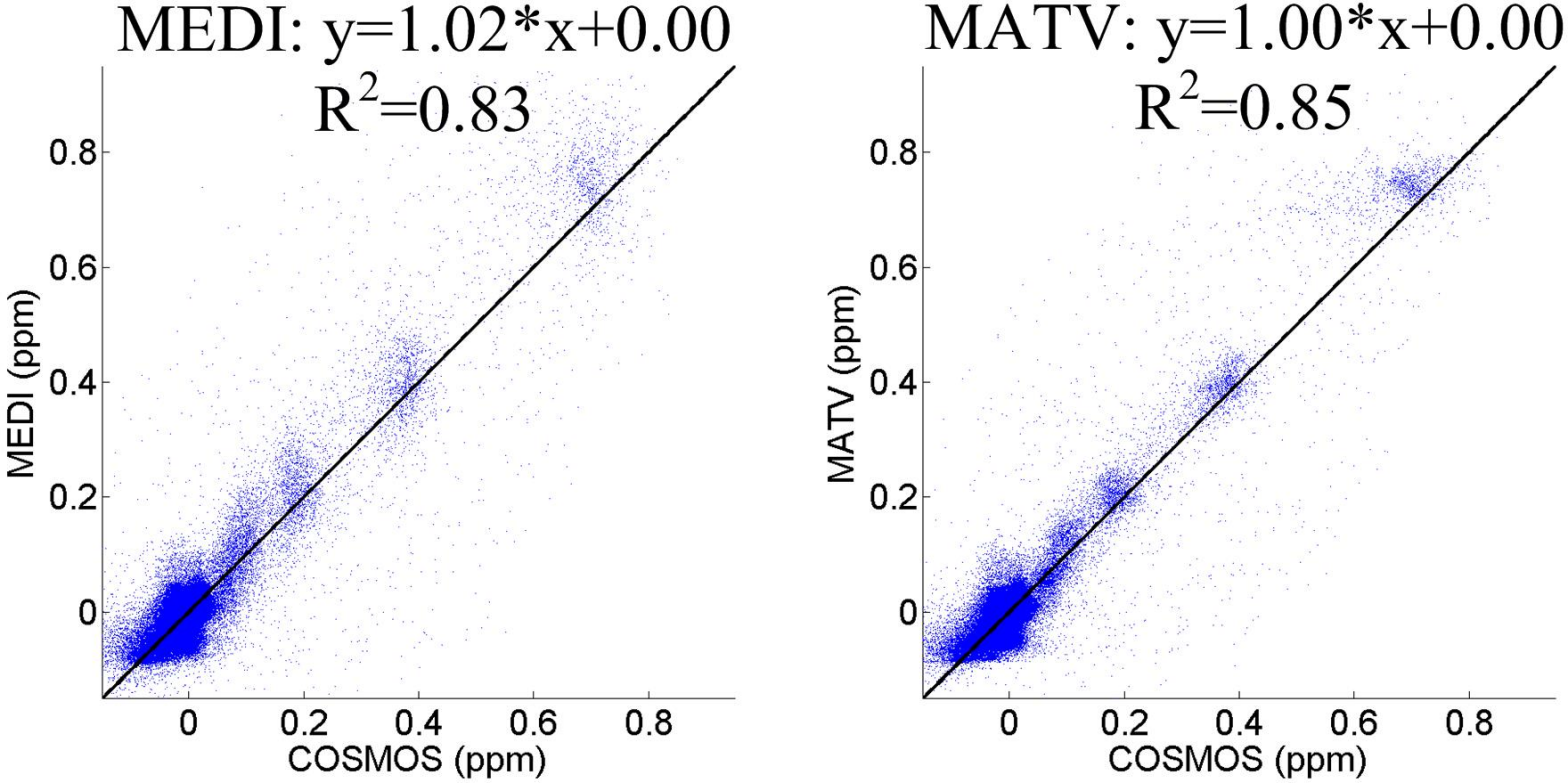
The linear regression analysis of MEDI and MATV for the phantom dataset. The susceptibility map determined by COSMOS was regarded as the reference.

Fig 1 shows the QSM results for the gadolinium phantom using MEDI and MATV, as well as the COSMOS. The susceptibility maps generated by MEDI contained obvious artifacts near edges. By contrast, MATV provided results with reduced artifacts near edges and increased homogeneous appearance in smooth regions. The absolute error maps of MATV and MEDI for the phantom dataset are shown in Fig 2. It can be seen that MATV yielded lower reconstruction error than MEDI near edges. Fig 3 exhibits the linear regression results of MEDI and MATV with respect to the COSMOS-generated susceptibility maps. It can be seen that MEDI and MATV provided susceptibility maps with comparable accuracy.

### In vivo human

**Fig 4:**
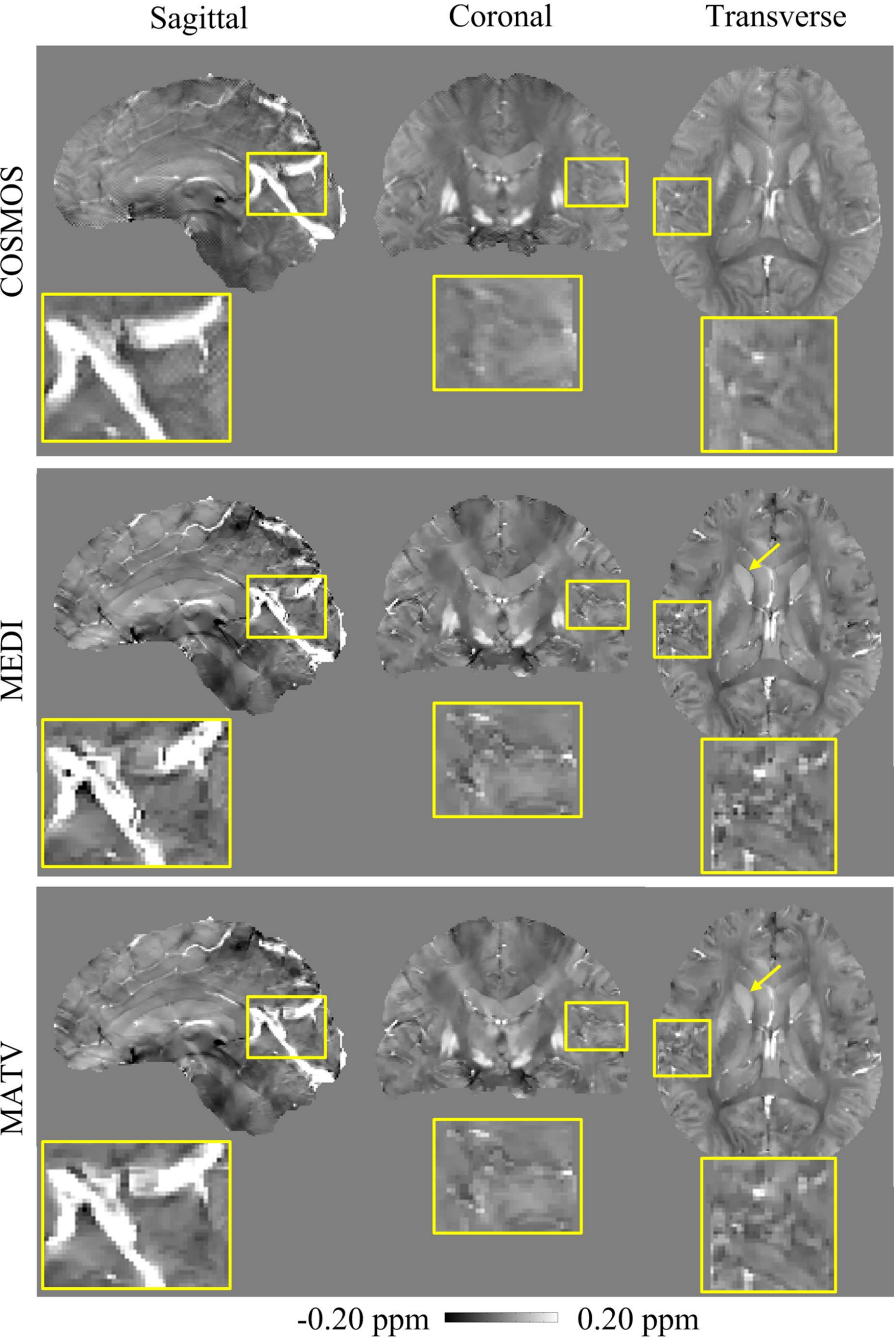
QSM reconstructions for the in vivo brain 1 dataset. First row: sagittal, coronal, and transverse views of susceptibility maps reconstructed by COSMOS. Second row: sagittal, coronal, and transverse views of susceptibility maps reconstructed by MEDI. Third row: sagittal, coronal, and transverse views of susceptibility maps reconstructed by MATV. The yellow box provides a zoomed-in view of each image.

**Fig 5:**
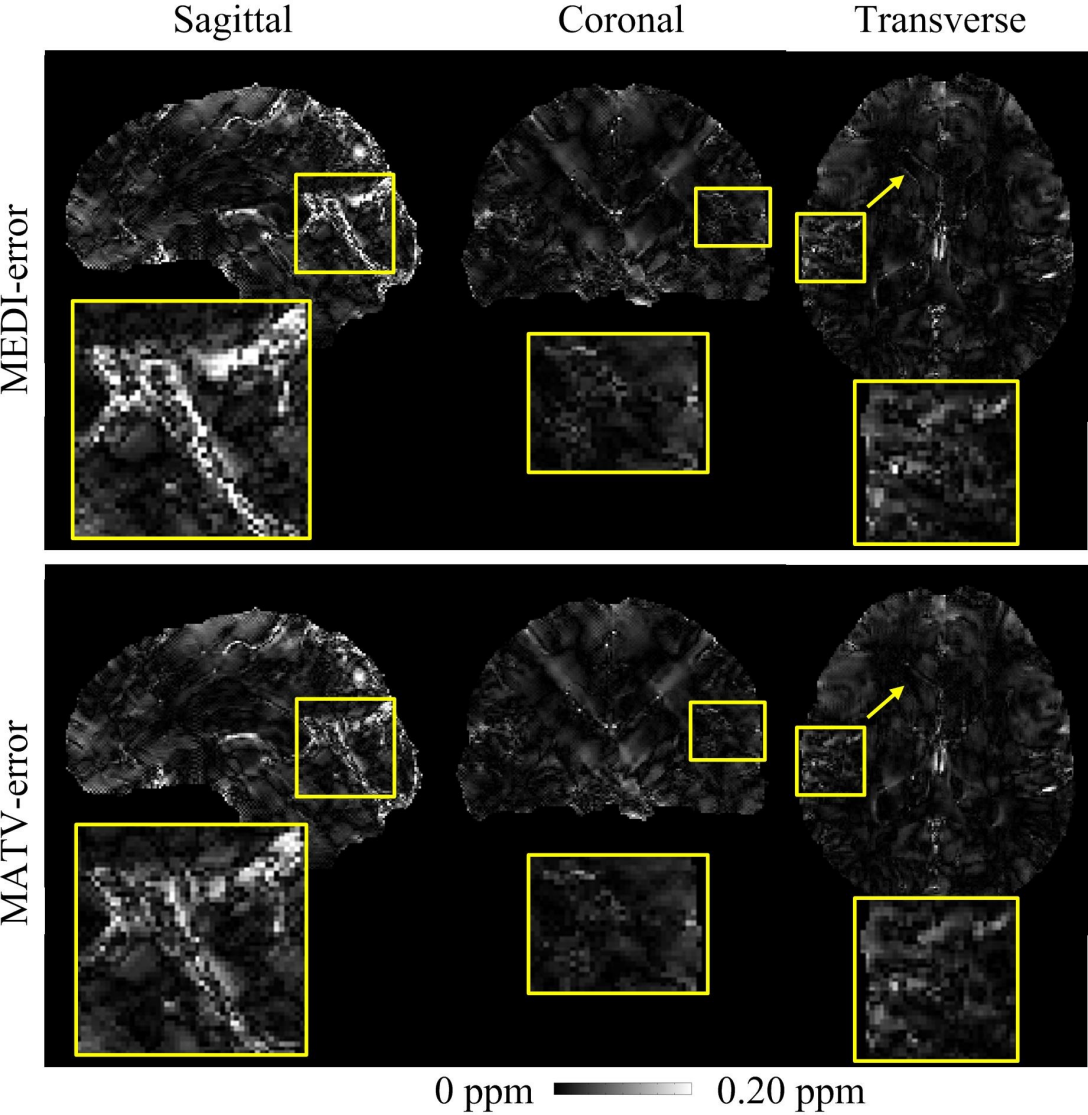
Absolute error maps for the in vivo brain 1 dataset. The susceptibility maps determined by COSMOS were regarded as the references. First row: sagittal, coronal, and transverse views of absolute error maps of MEDI. Second row: sagittal, coronal, and transverse views of absolute error maps of MATV. The yellow box provides a zoomed-in view of each error map.

The comparative results of MEDI and MATV using the healthy brain 1 dataset are presented in Fig 4, with the COSMOS results as references. The enlarged images show that the MEDI-generated susceptibility maps contain more artifacts near regions with tissue boundaries than those of MATV. The absolute error maps of MATV and MEDI for the in vivo brain 1 dataset are shown in Fig 5. It can be seen from the enlarged views that MATV yielded lower reconstruction error near edges than MEDI.

**Fig 6:**
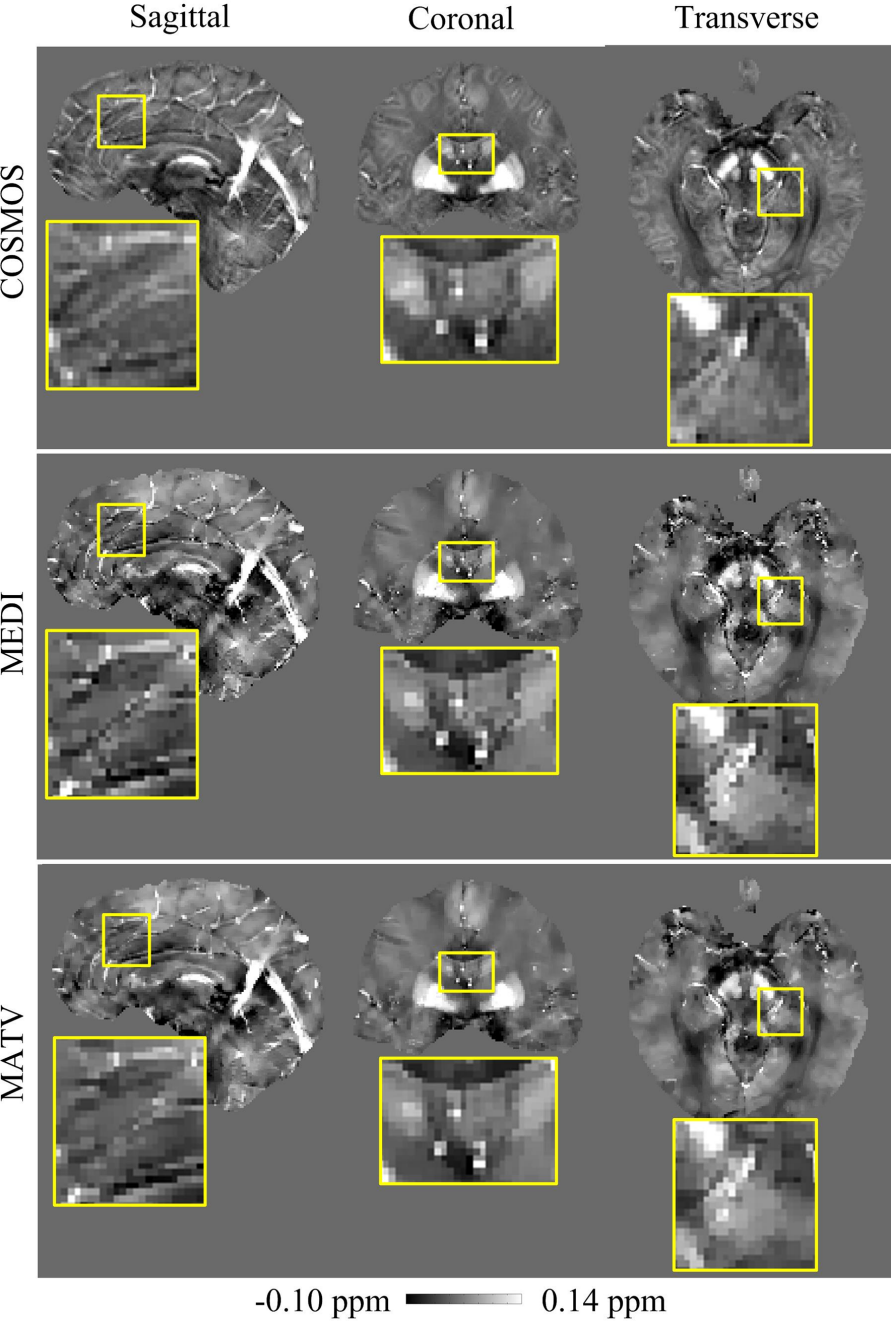
QSM reconstructions for the in vivo brain 2 dataset. First row: sagittal, coronal, and transverse views of reference susceptibility maps calculated by COSMOS. Second row: sagittal, coronal, and transverse views of susceptibility maps reconstructed by MEDI. Third row: sagittal, coronal, and transverse views of susceptibility maps reconstructed by MATV. The enlarged views indicate the regions where MATV outperformed MEDI.

**Fig 7:**
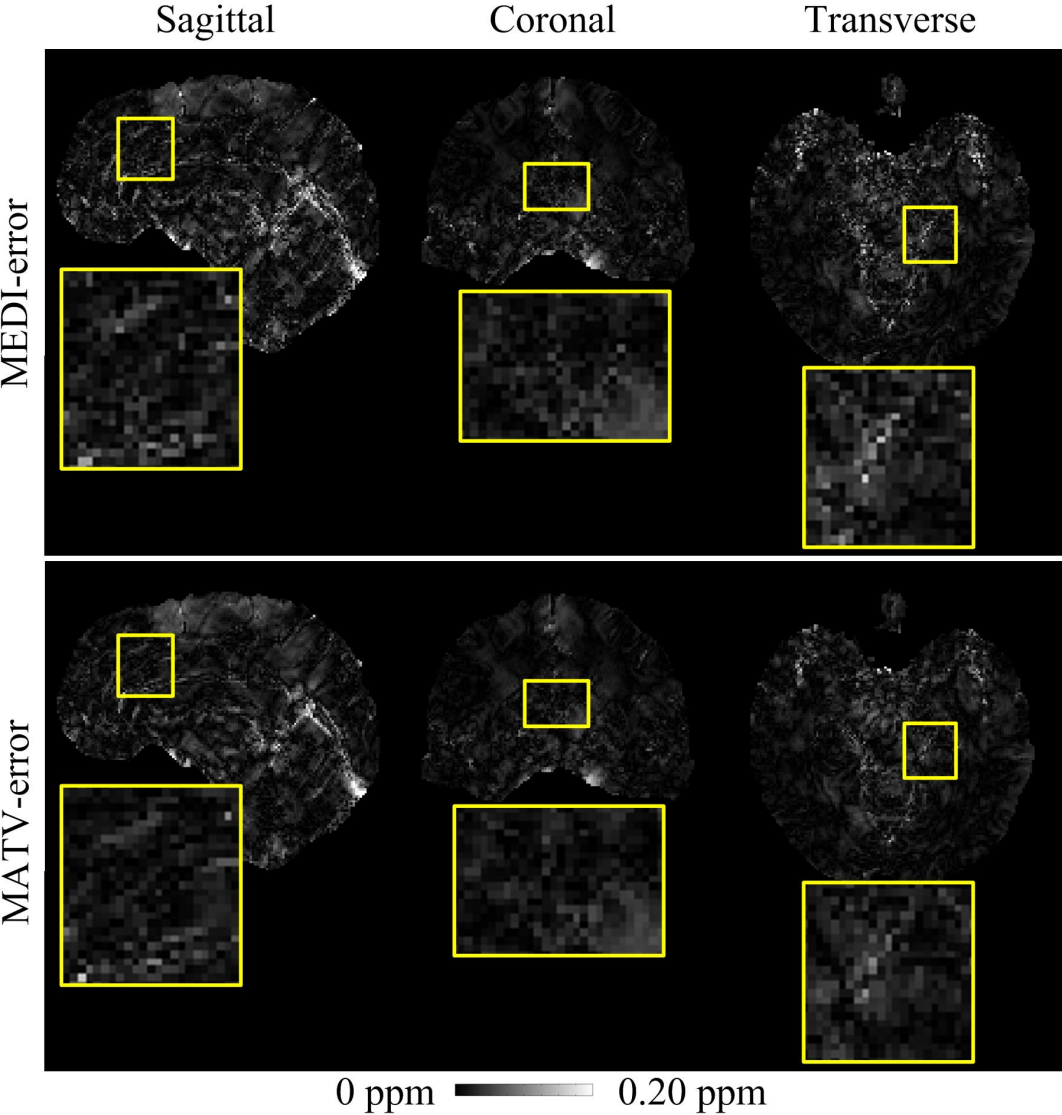
Absolute error maps for the in vivo brain 2 dataset. The susceptibility maps determined by COSMOS were regarded as the references. First row: sagittal, coronal, and transverse views of absolute error maps obtained by MEDI. Second row: sagittal, coronal, and transverse views of absolute error maps obtained by MATV. The enlarged views indicate the regions where MATV outperformed MEDI.

The COSMOS reference, MEDI, and MATV reconstructions for the healthy brain 2 dataset are shown in Fig 6. MEDI reconstructions exhibited substantial artifacts in regions with tissue edges (as shown in the enlarged views) compared with the reference COSMOS, and the artifacts were markedly reduced with MATV. Moreover, MEDI results appeared to be noisy inside smooth regions, but the noise was suppressed with MATV. The absolute error maps of MATV and MEDI for the in vivo 2 dataset are shown in Fig 7. It can be observed from the enlarged views that MATV yielded lower reconstruction error near boundaries than MEDI.

Table 1 summarizes the quantitative measures of RMSE, HFEN, and SSIM. The proposed MATV method produced lower RMSE and HFEN than MEDI for the phantom and the healthy human brain datasets.

**Table 1:** Accuracy comparison between the QSM reconstructions of MEDI and MATV.

		RMSE (%)	HFEN (%)	SSIM
Phantom	MEDI	70.17	74.06	0.98
	MATV	63.96	65.60	0.98
In vivo 1	MEDI	78.96	86.57	0.98
	MATV	74.19	79.31	0.96
In vivo 2	MEDI	84.44	75.17	0.91
	MATV	75.31	66.89	0.91

## Discussion

In this work, we developed a Morphology-Adaptive TV (MATV) regularization method for the reconstruction of tissue magnetic susceptibility map from single orientation MRI phase data. Compared with MEDI, the proposed MATV method demonstrated improved reconstruction quality near tissue edges in phantom and in vivo brain datasets.

Like MEDI, the current MATV method obtains prior information from magnitude image. One potential limitation of MATV is that the boundaries in magnitude image may be inconsistent with those in susceptibility map, and this inconsistency will cause errors in the reconstructed susceptibility maps. For example, putamen and white matter have weak contrast on the magnitude image, but strong contrast on QSM. Voxels in putamen and white matter will be penalized with strong TV constraints in MATV. As a result, the reconstructed susceptibility maps in putamen and white matter will be oversmoothed. To mitigate this problem, prior information from phase data or the reconstructed susceptibility maps themselves can be exploited [39, 40].

It is well known that it is a difficult task to optimize the regularization parameter in most inversion methods. Improper choice of parameter introduces either over-smoothness or streaking artifacts. In MATV, the regularization parameters were selected based on visual inspection and the quantitative metrics, i.e., RMSE, SSIM, HFEN, and regression slope. We firstly determined the range of regularization parameters by visual inspection. Then, we narrowed the range of parameters based on regression slope metric. Finally, the regularization parameters were selected based on other quantitative metrics, i.e., RMSE, SSIM, and HFEN. We tried the *L*-curve [16, 41] method to automatically select the regularization parameter at the point with the shortest distance to the origin. However, the parameters selected by the *L*-curve method produced lower regression slope values and more oversmooth susceptibility maps than those by the manual method. Other automatic parameter selection algorithms such as generalized cross validation [42] and Monte Carlo Stein's unbiased risk estimate [43] could be further investigated.

In conclusion, a MATV method for reconstructing susceptibility maps from the MRI phase was proposed. MATV imposed a TV constraint on the whole susceptibility map, and the regularization weights were a monotonically decreasing function of MRI magnitude gradients. Thus, smaller regularization degree was imposed on voxels in nonsmooth regions than those voxels in smooth regions. Phantom and in vivo imaging results demonstrated that MATV improves the reconstruction of susceptibility maps near tissue boundaries, and the image quality inside smooth regions may be improved as well.
